# Evaluating the international standards gap for the use of acupuncture needles by physiotherapists and chiropractors: A policy analysis

**DOI:** 10.1371/journal.pone.0226601

**Published:** 2019-12-17

**Authors:** Nadine Ijaz, Heather Boon

**Affiliations:** Leslie Dan Faculty of Pharmacy, University of Toronto, Toronto, Canada; University of South Australia, AUSTRALIA

## Abstract

**Background:**

Acupuncture needles have become an increasingly-popular treatment tool used by multiple health professions. However, the World Health Organization (WHO)’s 1999 training guidelines for acupuncture address only medical doctors and licensed acupuncturists, leaving a gap as to appropriate training standards for other professions.

**Aims and methods:**

With reference to an extensive document analysis, and interviews with seventeen acupuncture educators from across several professions in Ontario, Canada, this work uses a critical qualitative policy analytic approach to: a) present a comprehensive account of statutory training requirements for acupuncture-needling physiotherapists and chiropractors in the United States, Canada, and Australia; and b) evaluate competing stakeholder discourses pertaining to recent related controversies.

**Results:**

A wide range of educational requirements are evident across the jurisdictions under study (most below the 200-hour WHO guideline for physicians); and there is considerable disagreement among stakeholders as to what constitutes sufficient training in various forms of acupuncture, including ‘dry needling’. Organizations defending brief post-graduate training for needling physiotherapists and chiropractors are generally associated with these two professions, and contend that their ‘dry needling’ practices differ substantially from traditional acupuncture. Characterizing such brief training as insufficient, opportunistic and unsafe, and ‘dry needling’ as a subset of acupuncture practice, are the voices of all acupuncture educators interviewed, as well as professional organizations representing physicians, licensed acupuncturists, and some physiotherapists and chiropractors.

**Discussion and conclusion:**

Critiquing claims on both sides of the debate, this work calls for the development of independent, international safety-geared training guidelines that explicitly address the recent, evidence-informed trend towards biomedicalized acupuncture needling. Findings also suggest a need for additional research regarding the current shift towards overlapping—rather than exclusive—health professional practice scopes in industrialized countries.

## Introduction

First performed over 2500 years ago in China, acupuncture involves the therapeutic insertion and manipulation of thin needles at strategic body locations. The practice remains an integral component of several traditional East Asian medicine systems within and beyond China, where it is used to treat a wide range of health conditions [1]. Over the centuries, acupuncture has also spread to other continents, with European medical texts referring to the practice as early as the seventeenth century [2]. In recent decades, health care providers from across several occupational groups—most notably medical doctors, physiotherapists and chiropractors—have incorporated acupuncture needles into their therapeutic toolkits worldwide, often but not exclusively, to treat musculoskeletal pain [3]. Acupuncture needling approaches, also referred to as *trigger point dry needling* or *intramuscular stimulation*, are diversified within and across the health professions. Currently these therapeutic approaches may be based in East Asian medical theories, biomedical conceptions of the body, or a combination of the two. However, what unites this range of approaches is a common clinical use of ‘solid filament needle[s]’ [4, p. 134], a growing body of related bioscientific evidence [5], and increasing usage across the industrialized world.

Up from three million visits in 2001 [6], four million acupuncture sessions were given in the United Kingdom in 2009 [7]. These figures mirror an increase from 2.1 to 3.1 million adult patients in the United States who received acupuncture treatments between 2002 and 2007 [8]. Whereas 2% of Canadians had received treatment from an acupuncture practitioner in 2005 [9], usage among white and ethnic Chinese Canadians was reported as 8% in a 2008 study [10]. In a 2007 Australian study, 9% of respondents had similarly recently received acupuncture treatment [11]. As a health care intervention used with large numbers of patients, questions around patient safety invariably arise as policy makers negotiate appropriate parameters to govern the practice.

Quantifying acupuncture’s risks, several large prospective studies (see Table 1) [4, 12–18], evaluated in a recent systematic review [19, p. 4], have shown that ‘both minor and serious adverse events can occur from the use of acupuncture’. Minor adverse effects, which are characterized across this body of literature as common, include minor pain, localized bleeding and bruising. More severe effects, such as nausea, vertigo or fainting, headache, localized infection, disturbances in sleep, mood, hearing or vision, occur less frequently and typically resolve within a short time. Potentially-serious adverse events, such as organ puncture (e.g., pneumothorax), nerve injury, disease transmission, or cardiac tamponade, occur very rarely. As such, a general consensus exists that ‘acupuncture, in the hands of qualified practitioners is safe’ [16, p. 104].

**Table 1:** Overview of prospective acupuncture safety studies.

Citation	Health Professionals	Training Duration	Number of Treatments	Adverse Events^1^
Yamashita et al. 1998 [12]	Acupuncturists^2^ (n = 76), Japan	4 years F/T	55 291	*Minor*: 0.12% [P] *Serious*: 0.006% [P]
MacPherson et al. 2001 [14]	Acupuncturists (n = 574), United Kingdom	≥ 3 years F/T	34 407	*Minor*: 0.13% [T] *Serious*: None reported
Odsberg et al. 2001 [18]	Physiotherapists (n = 187), Sweden	Not specified	9277	*Minor*: 22.7% [T] *Serious*: None reported
White et al. 2001 [15]	Medical doctors (n = 47) and Physiotherapists (n = 30), United Kingdom	Not specified	31 822	*Minor*: 6.71% [T] *Serious*: None reported
MacPherson et al. 2004 [13]	Acupuncturists (n = 638), United Kingdom	≥ 3 years F/T	31 196	*Minor*: 10.7% [P] *Serious*: 0.05% [P]
Melchart et al. 2004 [16]	Medical doctors (n = 9429), Germany	All: ≥140 hours; 19% ≥ 350 hours	760 000	*Minor*: 7.1% [P] *Serious*: 0.006% [P]
Witt et al. 2009 [17]	Medical doctors (n = 13579), Germany	All: ≥ 140 hours; 15% ≥ 350 hours.	2.2 million	*Minor*: 8.6% [P] *Requiring treatment*: 2.2% [P]
Brady et al. 2014 [4]	Physiotherapists (n = 39), Ireland (Dry needling)	64 hours	7629	*Minor*: 19.18% [T] *Serious*: None reported

^1^as % of Patients [P], or % of Treatments [T]

^*2*^ The term ‘acupuncturist’ refers to acupuncture practitioners trained from an East Asian medical perspective, and whose training additionally includes biomedical content.

However, existing research has not established what constitutes sufficient training in the clinical usage of acupuncture needles to produce safe practitioners. Witt and colleagues [17] question—but do not resolve—the possibility of differences in the frequency or severity of acupuncture-related adverse events between different professional groups. Although some studies have examined acupuncture’s associated risks as delivered by specific professions as seen in Table 1, possible relationships between risk and training background remain underexplored. In a retrospective Australian study, Bensoussan and colleagues compared adverse event frequencies between acupuncturists with an East Asian medicine training (n = 642) and biomedical providers (n = 458); the latter group had significantly less acupuncture training on the whole (72% < two weeks). While the study authors identify higher per-treatment rates of adverse events reported by biomedical practitioners (0.27% vs. 0.1%), it is unclear whether to attribute this differential to training, under- or over-reporting practices, or some other factor(s) [20].

Regardless, acupuncture-related adverse effects have been characterized under two primary categories: those associated with standard practice, and negligence [21]. MacPherson and colleagues [13, p. 352] similarly note that a significant proportion of acupuncture-associated adverse events ‘might be considered avoidable… [as] they could only happen if acupuncture procedures were executed poorly’. Appropriate training, as such, may help to reduce the frequency and/or severity of negligent events.

Our aim in this work is to examine—using patients’ physical safety as a central focus—the regulatory acupuncture training standards currently implemented for chiropractors and physiotherapists in the United States, Canada and Australia. It should be noted that in Canada and the United States, chiropractors are widely recognized with a ‘doctor’ title; this is not the case in Australia. In none of the aforementioned jurisdictions do physiotherapists have a statutory ‘doctor’ title. This point will be taken up in greater detail further on.

Undertaken in the qualitative mode of critical policy analysis, and with reference to an extensive document review and seventeen semi-structured interviews, our approach in this work is three-fold. First, we review regulatory standards for physiotherapists and chiropractors with explicit or implicit statutory authority to use acupuncture needles within their treatment toolkits in the aforementioned countries. Second, we provide a descriptive overview of recent controversies surrounding the identified standards. Finally, we unpack these controversies by identifying and evaluating safety-related discourses around what constitutes sufficient acupuncture training for these providers. In light of these data, we provide a set of policy recommendations aimed at better protecting the increasing number of physiotherapy and chiropractic patients treated with acupuncture needles in industrialized countries.

Before turning to our study methods, we present some additional background.

### International training guidelines for acupuncture

In 1999, following an intensive consultative process involving ‘more than 50 international experts,’ the World Health Organization (WHO) released international ‘Guidelines for Basic Training and Safety in Acupuncture’ [1, p. 1]. This document was the first set of training benchmarks published by the WHO with reference to traditional and complementary medicine practices; the second, similarly detailing training guidelines for chiropractic, was published in 2005 [22]. A series of seven additional WHO training benchmarks in the traditional and complementary medicine field followed in 2010 [23], using a slightly-revised format. Although the WHO has not detailed training guidelines for physiotherapy, it has had a formal relationship with the World Confederation of Physical Therapy since 1952 [24, 25]; this organization has articulated its own set of basic professional training standards.

The WHO’s 1999 acupuncture guidelines specify recommended training parameters for four types of practitioners. Training guidelines for the first three of these practitioner groups is relatively straightforward, as follows: 1) ‘those with little or no prior medical education or experience’ seeking to practice as ‘traditional acupuncture practitioners’ (2500 hours); 2) ‘qualified physicians (modern Western medicine)’ seeking a ‘full training’ in acupuncture (1500 hours); 3) ‘qualified physicians …who wish to include acupuncture as a technique in their clinical work’ (‘not less than 200 hours’) [1, pp. 5–6].

For the fourth type of practitioner, ‘other health personnel (modern Western medicine)…working in the primary health care system of their country’ [p. 5], the WHO recommended trainings in *acupressure* rather than acupuncture. That said, the guideline noted that for ‘some personnel who show a particular aptitude,’ a ‘basic training in acupuncture’ might be arranged (p. 12); ‘basic’ training in the guideline elsewhere refers to the minimum of 200 hours recommended for physician practitioners (p. 10).

It is clear from the guidelines that all four aforementioned practitioner types, including acupuncturists trained in East Asian medicine, were recommended to have a substantive training in the biomedical sciences. The WHO furthermore recognized that many biomedically-trained providers would ultimately perform acupuncture from within a biomedical conceptual framework. That said, all training syllabi were substantively designed around Chinese medical explanatory models. The guidelines, the WHO noted, were

‘intended to assist national health authorities in setting standards and establishing official examinations, and also medical schools and institutions wishing to arrange training programmes’

(p. 1).

Formal examinations and statutory regulations associated with such acupuncture training programs would, the WHO proposed,

‘bring under control the situation, current in certain industrialized and developing countries, where commercial exploitation of acupuncture training and practice is not uncommon, with all the harmful consequences that may ensue’

(p. 5).

However, it remains historically unclear why the WHO did not directly address physiotherapists and chiropractors in its guidelines, given that acupuncture needles had already been introduced into these groups’ clinical toolkits, primarily for the treatment of musculoskeletal pain.

In 1984, for instance, the New Zealand Physiotherapy Board had formally recognized acupuncture as part of the profession’s scope [26]. Furthermore, in 1999, the same year the WHO released its acupuncture training guidelines, the World Confederation for Physical Therapy (an organization with links to the WHO) had formed the International Acupuncture Association of Physical Therapists. Similarly, as early as the mid- to late-1990s, chiropractors in the United States were involved in interprofessional struggles over acupuncture [27]; and WHO staff, who co-ordinated development of basic professional training guidelines for chiropractic practitioners, would certainly have been aware of that occupation’s modes of practice.

Regardless, the WHO’s acupuncture guideline does not explicitly mention chiropractors or physiotherapists; nor does the WHO’s 2005 Training Guideline for Chiropractic refer to the practice of acupuncture. Although some jurisdictions grant statutory ‘doctor’ titles to chiropractors, they are not referred to as physicians (but rather as ‘traditional and complementary/alternative medicine’ providers) in the WHO’s *chiropractic* training guideline. Nor are chiropractors universally regulated as ‘physicians’ across nations, as the case of Australia makes clear. The WHO moreover explicitly specified its use of the term “physician” to mean “qualified physicians (modern Western medicine)” in its 1999 acupuncture guideline. While today’s chiropractic profession relies increasingly on biomedical perspectives, it has historically relied on a distinct, non-biomedical set of diagnostic perspectives (e.g., vertical subluxation of the spine). Regardless of individual nations’ regulatory parameters, it may thus be inferred that the WHO’s acupuncture guideline intended to characterize chiropractors not as physicians, but rather as practitioners to whom it would be preferable to teach *acupressure*. For this reason we interpret the WHO’s use of the term ‘physician’ as intended to refer to biomedical doctors, and not to the wide range of other health professionals (e.g., osteopaths, nurse practitioners, naturopaths, pharmacists) who may be granted ‘doctor’ titles by either institutions or governments across the globe.

In short, the WHO’s 1999 training guidelines for acupuncture have left a significant international gap with respect to a range of allied health professionals’ increasing adoption of this practice within their respective scopes. It should be noted that in a 2010 benchmark document outlining training parameters for Traditional Chinese medicine practitioners, the WHO refers to its 1999 acupuncture guideline as authoritative, not proposing any changes to the standards.

### Regulatory uptake of the World Health Organization’s acupuncture standards

Regulatory training requirements for East Asian medicine practitioners across the globe adhere roughly to the WHO’s 2500-hour standards [see 28]. Across the United States, Canada and Australia where traditional acupuncturists are widely subject to statutory regulation, a minimum of three years of full-time training are required for professional licensure in most jurisdictions (i.e., approximately 2000 hours) [29–31]; that said, these jurisdictions apply a wide range of regulatory models. However, regulatory uptake of the WHO’s 200-hour training guidelines varies considerably across the industrialized world for medical doctors who use acupuncture as an adjunct modality.

In Australia, where physicians may receive state reimbursement for delivering acupuncture treatments [32], trainings required for these physicians to use the protected title of ‘Registered Acupuncturist’ adhere to the 200-hour WHO guideline, and include a written and clinical examination [33]. In Canada, some provinces (e.g., Quebec) articulate minimum training requirements (exceeding the WHO standard) for acupuncture-practising physicians [34], whereas elsewhere (e.g., Yukon territory) no such training requirements are stipulated [35]. A majority of American states permit medical doctors to perform acupuncture, eleven among which stipulate training requirements approximating (or slightly exceeding) the 200-hour WHO guidelines. Three states require physicians to complete full-length acupuncture trainings on par with licensed acupuncturists [36].

In the absence of formal regulatory standards in many jurisdictions, voluntary acupuncture certification standards for physicians have been introduced in both Canada and the USA. In particular, the standards of American Board of Medical Acupuncture slightly exceed the WHO’s recommended standard at 220 hours of training plus two years clinical experience [37]. This organization describes itself as having the highest standards of training and proficiency among physicians practicing in North America (p. 1), affirming that the level of training received by acupuncture-practising medical doctors does indeed vary.

### Acupuncture training guidelines for physiotherapists and chiropractors

As noted earlier, no international consensus exists as to what constitutes sufficient training for physiotherapists or chiropractors using acupuncture needles to treat patients. Across the globe, professional associations and voluntary self-certification bodies have articulated a wide range of acupuncture-related training guidelines for these health professionals. The International Acupuncture Association of Physical Therapists—whose parent organization, the World Confederation for Physical Therapy, has a long-standing affiliation with WHO—advises ‘a basic training of 80 hours of relevant study in line with the WHO recommendations’ [38, p. 6]. No specification of how this guideline aligns with the WHO’s 200-hour basic training standard is provided, although the document refers to traditional acupuncture, Western medical acupuncture, and dry needling as related practices. In the United Kingdom, for example, a voluntary certification body calls for physiotherapists to ‘undergo a minimum of 300 hours of acupuncture training’ [39, p. 1]; a similar New Zealand association requires 150 training hours among its members [26]. The Irish Society for Chartered Physiotherapists, by contrast, stipulates a minimum 21 hours of training for those who perform ‘dry needling’ with patients [40, p. 2].

In 2006, the Australian Society of Acupuncture Physiotherapists recommended two distinct minimum voluntary training standards: 150 hours for those learning traditional acupuncture, and a ‘2 day course’ for those using acupuncture needles to perform ‘Dry Needling or Western Acupuncture’ [41, p. 3]. Seven years later, the same organization reduced its recommended number of training hours for traditional acupuncture by almost half, to 80 hours; although its stipulated guideline for ‘western acupuncture’ or ‘dry needling’ on the basis of ‘clinical reasoning’ underpinned by ‘anatomical and neurophysiological knowledge’ remained constant at 16 hours [42, p. 8].

In the United States, where the term *dry needling* has widely been adopted to describe physical therapists’ clinical use of acupuncture needles, the Federation of State Boards of Physical Therapy has produced an ‘analysis of competencies for dry needling’ [43, p. ii], in which it states that ‘dry needling is not an entry-level technique’, thereby warranting some postgraduate ‘specialized training’ (p. 12). Citing lack of consensus as to ‘the minimum number of practice hours necessary’ (p. 13), the Federation has articulated no specific minimum training standards for the practice.

With respect to chiropractors, there are few formally-articulated training guidelines for acupuncture; however, where they exist, such guidelines vary notably. In the United States, for instance, two different organizations have stipulated distinct sets of standards. The National Board of Chiropractic Examiners [44] offers a voluntary acupuncture examination to certify chiropractors who have documented a minimum of 100 hours of acupuncture training. The American Board of Chiropractic Acupuncture, by contrast, certifies chiropractors in acupuncture on the basis of a 300-hour training accompanied by an examination [45].

We are unaware of any studies to date that document the risks associated with acupuncture needling performed by chiropractors who, like physiotherapists, generally use the practice to treat musculoskeletal conditions within their broader scope. However, as seen in Table 1, adverse events are certainly evident among acupuncture-needling physiotherapists (as they are among medical doctors and traditionally-trained acupuncturists), raising policy considerations. As Johnson and colleagues document in the New Zealand context, 3.6% (n = 10 of 279) of all physiotherapy-related insurance injury claims from 2005 to 2011 were acupuncture-related; three of these ten claims were noted to be of ‘major’ harm consequence to the involved patient.[46] ‘Major’ harm in that context was defined as ‘short-to-medium lessening of bodily function…unrelated to the natural course of the illness’ (p. 70); no deaths or major permanent injuries were reported. It is evident that additional research is needed to investigate what constitutes sufficient training for such providers, to minimize the risk of unnecessary adverse events.

## Study aims and methods

This study was approved by the University of Toronto Research Ethics Board, Protocol #30416. Our aims in this work are three-fold: 1) to review statutory training standards for physiotherapists and chiropractors who use acupuncture needles in the United States, Canada and Australia; 2) to provide an overview of recent controversies related to these regulatory standards; and 3) to characterize and analyse the primary discourses that characterize ongoing debates among various stakeholders as to what constitutes sufficient training for these providers. To meet these study aims, as detailed below, we generated a body of data from a) a compilation of documents, and b) seventeen semi-structured interviews. In the methodological mode of critical policy analysis, we then analysed and interpreted this qualitative dataset using a combination of descriptive content analytic and critical discourse analytic approaches.

### Document compilation

To provide the basis for our analysis, we compiled a dataset of public and scholarly documents addressing regulatory and certification standards for the use of acupuncture needles by chiropractors and physiotherapists across the English-speaking, industrialized world. Citations to all core analysed documents are provided within this paper’s text.

First, we compiled an exhaustive list of acupuncture-related statutory standards for physiotherapists and chiropractors in Canada, the United States and Australia. These three jurisdictions were selected on the basis that they are English-speaking countries in which regulatory standards are widely in place to govern the practice of acupuncture by TCM practitioners as well as physicians, physiotherapists and chiropractors. To meet this aim, we used an online search strategy to collect standards-related documents from government / regulator websites, as well as professional associations and certification agencies associated with each of the aforementioned professions. In some cases, in which particular standards had been entrenched or overturned by court rulings, we referred directly to court documents and government consultative transcripts. Although media reports provided preliminary information about some of these court rulings, only data confirmed from a regulatory source, professional association or certification agency were included in the study. Data about standards were extracted from each of these documents and presented in table and narrative form.

The second documentary aim was to compile documents that explicitly addressed recent controversies surrounding the aforementioned standards across the countries under study. In addition to reviewing all previously-identified, standards-related documents to this end (see above), we identified the names and websites of additional professional organizations, associations and certification bodies associated with the use of acupuncture needles by chiropractors, physiotherapists, East Asian medicine practitioners and medical doctors in English-speaking countries. We searched each of these organizational sites for documents that include position statements that addressed needling practices. Such position statements were extracted in full into Nvivo^™^ for analysis, detailed further on.

### Semi-structured interviews

As part of her doctoral research, the first author conducted thirty-three semi-structured interviews with a range of stakeholders involved in the statutory regulation of acupuncture and traditional Chinese medicine (TCM) in the province of Ontario, Canada over the period 2013–2017. Additional details about the entire project may be found in the published dissertation [47]. In Ontario, significant controversy has surrounded acupuncture training standards implemented for members of the ten regulated professions authorized to perform acupuncture within their statutory scope in 2013. (These professions are: chiropractic, chiropody, dentistry, massage therapy, medicine, naturopathy, nursing, occupational therapy, physiotherapy, and traditional Chinese medicine [48].) Approval to conduct these interviews with adult participants was granted by the University of Toronto’s Research Ethics Board.

Study participants, who included health care professionals as well as government regulators, were recruited on the basis of their public involvement in Ontario’s acupuncture and TCM regulatory process. Names of prospective participants were identified in government consultations, reports and transcripts, as well as media reports pertaining to the regulations under study; and by direct referrals from other study participants. Persons who had not been directly or indirectly involved with the regulatory process under study were excluded from eligibility to participate. In total, fifty-five persons were invited to be interviewed, of whom thirty-three (60%) consented to participate. Interview invitations were delivered by e-mail or lettermail, and followed up on two occasions when no response was received.

Based on a semi-structured interview guide addressing key study themes (attached as S1 Table), 60–90 minute interviews were conducted concurrently with analysis towards saturation of the range of participant perspectives [49]. The issue of professional entry standards was among the pre-determined themes addressed in the interview guide. Recruitment addressing this theme continued until the discourse analytic process (detailed below) produced no additional results. The majority of interviews (and all those included in the present study) were conducted face-to-face (rather than by telephone).

Following a written informed consent process, all interviews were audio recorded and verbatim transcribed for analysis. Interview transcripts were entered into NVivo^™^ qualitative analytic software to facilitate a process of rigorous and transparent coding. After preliminary review of the entire interview set, and in consultation with the first author’s PhD supervisor (the second author), a subset (n = 17) of interviews with acupuncture educators was selected for deeper analysis in relation to the present study aims.

Acupuncture educators, we postulated, might have particular experience and insight surrounding acupuncture-related training, addressing considerations of practitioner safety and proficiency. The interviewed educators (eleven male, seven female) were all regulated professionals, representing the professions of TCM, medicine, physiotherapy, chiropractic, and massage therapy. Five interviewees exclusively delivered acupuncture education courses for non-TCM professionals, two taught both TCM and non-TCM professionals, and ten only trained TCM professionals. Each of the seventeen interviews included semi-structured questions about the participant’s experience as an acupuncture educator, as well as her or his views about the Ontario’s statutory training requirements for acupuncture.

It should be noted that while the present study conducts documentary analyses pertaining to three countries, the interviews included in the study represent a case study from a single jurisdiction, meant to triangulate and nuance findings gleaned from documentary data. In qualitative case study methodology, it is accepted that singular localized cases may be used ‘instrumentally’ to produce generalizable insights pertaining to a broader research question [50]. The decision to select for inclusion in the present study a subset of interviews with acupuncture *educators* in particular was inductively made in light of the rich, standards-focused data that emerged in these particular interviews during preliminary data coding of all interviews.

### Analysis and interpretation

Centralizing our study aims, we undertook analysis of our dataset in the mode of critical policy analysis. As Howarth [51, p. 324] notes, ‘[t]he aim of critical policy studies is to critically explain how and why a particular policy has been formulated and implemented, rather than others’. The analytic process begins by ‘*problematizing* a particular policy, practice or regime’ (p. 324), typically by critiquing the way a particular policy problem is constituted by stakeholders. Next, data are engaged to provide an explanation for the emergence and/or persistence of the problematized policy, as well as its broader social, political or ideological purposes. Finally, the critical policy analyst is charged with ‘reactivat[ing] those options that were foreclosed during the emergence of a practice or policy’ (329). As Diem and colleagues [52] advise, qualitative research modes are often better suited to critical policy studies than are quantitative analyses.

In this work, we apply two primary qualitative analytic approaches to our dataset: descriptive content analysis and critical discourse analysis. Descriptive content analysis [53]–applied in the first two phases of our analysis—is a qualitative approach that emphasizes description rather than critical interpretation.

Our first analytic phase presents an exhaustive descriptive account of statutory training requirements implemented for acupuncture/dry needling, as performed by physiotherapists and chiropractors, across the United States, Australia and Canada. These training requirements were progressively compiled, in table form, from across the collected documents. Documents published by regulatory bodies and professional organizations were the primary sources of the data compiled in this phase.

Because the regulatory status of acupuncture needling by physiotherapists and chiropractors has recently changed in some jurisdictions, documents were cross-referenced to ensure up-to-date reporting. Where gaps or inconsistencies were evident, the first author corresponded directly (by e-mail or telephone) with jurisdictional regulators in an effort to complete an exhaustive account. For a small number of jurisdictions, our efforts to procure relevant data were unsuccessful; this is noted explicitly in our study results.

The aim of the study’s second analytic phase was to compile an account of recent and ongoing controversial public activity pertaining to acupuncture needling standards for chiropractors and physiotherapists in the United States, Canada and Australia. Using NVivo^™^ qualitative analysis software, and in ongoing corroboration with the second author, the first author analysed the compiled documents and interview transcripts using thematic analytic methods as described by Braun and Clarke [54]. Thematic analysis is characterized by a process of textual coding and categorization that reviews and progressively characterizes recurrent or salient features evident in the texts under study. The results of this analysis are presented in narrative form in this work’s results section.

The descriptive data presented in phases one and two provide important context for our more critical analytic work in phase three, which proceeds in the mode of critical discourse analysis (CDA). As Fairclough [55] notes, CDA—a common approach in critical policy studies—seeks to expose the conceptual underpinnings and power relations submerged in the linguistic content and form of particular texts. Beginning with a thematic analytic approach similar to that undertaken in phase two, the first author reviewed the compiled documents and interviews to identify verbatim textual excerpts representing the range of stakeholder perspectives pertaining to statutory training standards for acupuncture needling physiotherapists and chiropractors in the three nations under study. As this analysis progressed, it became clear that patient safety was a primary recurrent theme across stakeholder accounts; and, while other issues were evident, we made a decision to focus our analysis primarily on risk-related discourses.

Interpreting stakeholder perspectives as politicized discourses rather than neutral themes, the authors then sought to further analyse and interpret these excerpts in line with Bacchi’s policy-focused CDA methodology [56]. This approach critically interrogates the discursive representation of particular policy problems by various stakeholders, whether explicit or implicit, giving attention to broader sociocultural, economic and political contextual features at play. These interpretive findings are presented—using supportive verbatim textual excerpts—as phase three of our analysis; and are deployed along with descriptive findings from phases one and two in the study’s Discussion and Conclusion section, to meet the broader aims of critical policy analysis (described earlier on).

### Rigour and reflexivity

Four primary principles—credibility, dependability, transferability and confirmability—have been differentiated in the literature as essential in demonstrating rigour in qualitative research; and each of these principles may be variously demonstrated in practice [57, 58]. Our use of multiple data sources (documents and interviews) enhances this work’s credibility and dependability. Our use of NVivo^™^ software throughout the analytic process, producing an audit trail, contributes to the study’s dependability and confirmability; and thick analytic descriptions supported by raw data (e.g., in tables and verbatim quotes) confer transferability upon the presented findings. Our explicit articulation of the study’s geographic and thematic (i.e., safety-related) boundaries moreover support’s this work’s transferability.

The principle of researcher *reflexivity* has, in addition, been widely characterized as ‘a means to enhance the rigo[u]r of the study and its ethics’ [59, p. 221]. Reflexivity refers to research conditions under which those conducting research attend consciously and explicitly to their subjective engagement with research paradigm, subject matter and participants. Most notable in the present context is the co-authors’ long-standing professional engagement in the fields under study, contributing to the study’s credibility.

Both authors are policy researchers in the field of traditional, complementary and integrative medicine. While the first author has undergone formal training in traditional acupuncture, she has not performed the practice professionally. The second author has a long-standing scholarly involvement with health professionals across multiple fields and is herself a licensed health care professional (pharmacist). The first author’s personal familiarity with acupuncture practice assisted in building a rapport with study interviewees, and in characterizing key controversies identified in our study findings. Both authors’ familiarity with the professional cultures surrounding acupuncture helped to inform the analysis presented in this work.

## Results

### Part I. Acupuncture training requirements for chiropractors and physiotherapists in the United States, Canada and Australia

#### United States

As seen in Table 2, chiropractors are permitted to use acupuncture needles in a majority of American states (n = 35). They generally describe this practice as *acupuncture* except where the term is exclusively authorized to licensed acupuncturists; there, they have adopted alternate terminology (in three states, *dry needling* and in one state, *meridian therapy*). In five of thirty-five states, no related training standards are stipulated; and two others require fewer than 100 training hours. Another eighteen states require 100 hours of acupuncture training, among which seven require successful completion of the National Board of Chiropractic Examiners’ acupuncture examination. Ten other states require acupuncture training at or above the WHO’s 200-hour standard articulated for physicians.

**Table 2:** Statutory acupuncture needling training requirements for chiropractors in the United States, Canada and Australia.

Training required	United States [45]	Canada^a^ [34, 60, 61]	Australia [62]
*Permitted*, *but specific training requirements not stipulated*	Connecticut, Kansas, Maryland (DN^b^), New Hampshire (DN), New Mexico (MT^b^)	Manitoba, Alberta, Saskatchewan	All jurisdictions (DN/MT)
*< 100 hours*	Louisiana (DN), Massachusetts		
*100 hours*	Alabama (E^c^), Arkansas, Arizona, Colorado, Delaware, Florida (E), Illinois, Iowa, Minnesota (E), Missouri (E), Nebraska, North Dakota, Oklahoma, South Dakota (E), Texas, Utah, West Virginia (E), Wyoming (E)		
*200–250 hours*	Idaho, Indiana, Maine, North Carolina, Virginia, Washington DC (E)	New Brunswick, Ontario, Nova Scotia	
*300 hours training*	Alaska, Ohio, Tennessee (E), Vermont		
*Not permitted without full acupuncture license*	California, Georgia, Hawaii, Kentucky, Michigan, Mississippi, Montana, New Jersey, New York, Nevada, Oregon, Pennsylvania, Rhode Island, South Carolina, Washington, Wisconsin	British Columbia, Quebec	
Status unknown/unclear		Newfoundland/Labrador, Prince Edward Island, Yukon	

^a^ Chiropractic not regulated in Northwest Terrotories, Nunavut

^b^ DN, MT = Use ‘dry needling’ or ‘meridian therapy’ rather than ‘acupuncture’ terminology

^c^ E = 200 question multiple choice National Board of Chiropractic Examiners (2015) examination required

Physiotherapists (called *physical therapists* in the USA) are currently using acupuncture needles within their clinical scope in over two-thirds of American states (n = 36), in most cases under approval by their state practice boards rather than by legislative sanction. American physical therapists universally refer to their use of acupuncture needles as *dry needling*. In 60% of states (n = 22) in which physical therapists may perform dry needling within their scope, regulators have stipulated no minimum training standards, as seen in Table 3. Among those states (n = 13) that have implemented specific dry needling standards for physical therapists, all but one stipulate that practitioners document a minimum amount of postgraduate training that is less than 100 hours.

**Table 3:** Statutory acupuncture needling training requirements for physiotherapists^a^ in the United States, Canada and Australia.

Training required	United States (DN^b^) [63, 64]	Canada [34, 65–72]	Australia (DN) [73]
*Permitted*, *but specific training requirements not stipulated*	Alabama, Arkansas, D.C., Illinois, Indiana, Kansas, Kentucky, Nebraska, Nevada, New Hampshire, New Mexico, North Carolina, North Dakota, Ohio, Oklahoma, Rhode Island, South Carolina, Texas, Vermont, West Virginia, Wisconsin, Wyoming	Alberta, Ontario, Newfoundland/ Labrador, Quebec (DN), Yukon	All jurisdictions
*< 100 hours*	Alaska, Arizona, Colorado, Delaware, Georgia, Iowa, Louisiana, Maryland, Mississippi, Montana, Tennessee, Utah, Virginia		
*100–200 hours*		Manitoba, Nova Scotia	
*200–250 hours*	Maine	British Columbia, New Brunswick, Prince Edward Island, Saskatchewan	
*Not permitted without full acupuncture license*	New York, Idaho, Florida, Hawaii, California, New Jersey, Pennsylvania, South Dakota, Washington		
*Status unknown/ unclear*	Connecticut, Massachusetts, Michigan, Minnesota, Missouri, Oregon	Northwest Territories, Nunavut	

^a^ Termed ‘physical therapists’ in the United States

^b^ DN = Use ‘dry needling’ rather than ‘acupuncture’ terminology

#### Canada

Chiropractors and physiotherapists are permitted to use acupuncture needles in a majority of Canadian provinces and territories. The term *acupuncture* is in widespread use by regulators governing both professions, except in the province of Quebec, where physiotherapists may only use *dry needling* terminology. Half of Canadian jurisdictions permitting chiropractic acupuncture stipulate no related training standards, whereas the remainder require 200 hours of training. Standards for physiotherapists vary more, with five provinces and territories articulating no specific training requirements, two requiring 100–200 hours, and four adhering to the WHO’s 200-hour guideline for physicians.

#### Australia

Under a national health professional regulatory model, the Acupuncturist title has since 2012 been exclusively restricted to those Australia’s regulated professions which have applied for a statutory *endorsement* to perform acupuncture within their scope. Each *endorsed* profession has authority to set unique acupuncture training requirements for its members, who may then use the Acupuncturist title. To date, only two professions have applied for the option of acupuncture endorsements: Chinese medicine practitioners and physicians. Several other Australian professions, including chiropractic and physiotherapy, have explicitly indicated that they are not opting into the acupuncture endorsement model.

In this statutory environment, members of any professions (and, in fact, members of the public) may freely use acupuncture needles, as long as they avoid use of *acupuncture* terminology. In other words, there are currently no statutory training requirements to govern what is widely referred to as *dry needling* among Australia’s chiropractors and physiotherapists.

### Part II. Controversies surrounding the use of acupuncture needles by chiropractors and physiotherapists

#### United States

The range of standards implemented for the use of acupuncture needles by chiropractors and physical therapists across the United States has proven significantly contentious in recent years. Several professional organizations representing medical doctors, licensed acupuncturists and chiropractors have articulated—in a series of position statements—their objections to existing regulatory parameters surrounding the use of acupuncture needles by American chiropractors and physiotherapists.

These organizations include: the American Medical Association [74], American Academy of Medical Acupuncture [75], American Academy of Physical Medicine and Rehabilitation [76], American Society of Acupuncturists [77], National Certification Commission for Acupuncture and Oriental Medicine [78], American Association of Acupuncture and Oriental Medicine [79], the American Traditional Chinese Medicine Association [80], Council of Colleges of Acupuncture and Oriental Medicine [81], and the Council of Chiropractic Acupuncture [82]. Whereas most organizations call for an increase in these providers’ training requirements, generally to the 200-hour WHO standard, some stand for the outright exclusion of acupuncture needles from within chiropractors’ and physical therapists’ scope.

In several American states, licensed acupuncturists have furthermore taken to the courts and legislatures—largely unsuccessfully—to contest chiropractors’ and physical therapists’ existing or prospective jurisdictional claims to use of acupuncture needles. Their stance is that in many cases, acupuncture needling does not fall within the statutory practice scopes of physiotherapists or chiropractors, rendering the practice illegal. Such appeals were recently successful in three instances. In 2014, in the case of *Oregon Association of Acupuncture and Oriental Medicine v*. *Board of Chiropractic Examiners*, Oregon chiropractors’ authority to perform *dry needling* (with twenty-four hours of postgraduate training) was denied on the basis that it was outside of the profession’s statutory scope [83]. More recently, in 2016, Washington state senators narrowly defeated a bill to expand physical therapists’ scope to include dry needling (with fifty-four hours of training) [84, 85]. Finally, New Jersey’s Attorney General issued a 2017 opinion that dry needling and intramuscular stimulation, practices that involve acupuncture needles, do not fall within the scope of the physical therapy profession, putting an end to the practice there [86].

#### Canada

The most notable and prolonged controversy around the use of acupuncture by Canadian chiropractors and physiotherapists has taken place in the province of Ontario, where the practice of acupuncture was regulated (and thus removed from the public domain) in 2013. The question of which regulated professions should be authorized to use acupuncture needles, using what terminology, and with what training standards, was contentious in that province over the years prior to regulation.

As elsewhere detailed [see 87], in 1996, the Ontario government initially recommended that delivery of acupuncture treatments should be limited to those with training in traditional acupuncture, but that other professions be permitted to use acupuncture needles but use alternate terminology to describe the practice. By 2001, the government modified its stance and recommended that in addition to members of a proposed Chinese medicine profession, dentists, physicians, nurses and naturopaths who met the WHO’s 200-hour basic training guideline be permitted to perform acupuncture, regardless of the medical framework (i.e., traditional or biomedical) used. Under this proposal, other professions—such as chiropractic and physiotherapy, which had expressed a vigorous interest in securing statutory access to acupuncture needles—would be required to petition for a specific expansion to their statutory scope and establish appropriate training standards.

By 2005, after vigorous lobbying on the part of these two professions, legislation was drafted that would ultimately (in 2013) permit chiropractors and physiotherapists, amongst other Ontario professions, to perform acupuncture within their respective scopes. Aligned with the province’s self-regulatory model, occupationally-specific training standards would be established by each profession’s regulatory body. Throughout the regulatory process, vocal objections to this proposal were evident from the province’s Chinese medicine practitioners. who variously argued that such a regulatory framework would compromise patient safety, result in delivery of ineffective care, and harm their own ability to make a viable income. Whereas Ontario’s chiropractic profession today requires its members to complete 200 hours of acupuncture training to perform the practice [61], the amount of acupuncture training needed for Ontario physiotherapists is left to the individual practitioner’s discretion [68]. The province’s professions of medicine, nursing and dentistry have similarly articulated no acupuncture-related training standards for their members.

There have been few public objections to this turn of events since the regulations came into effect. However, qualitative interviews undertaken by our research team confirm that traditional acupuncture practitioners, as well as acupuncture educators from across several regulated health professions, object strongly to the province’s current regulatory parameters on safety-related grounds. Our analysis further on will refer specifically to interviews with Ontario acupuncture educators.

#### Australia

As detailed by Janz and Adams [88, p. 3], Australia was ‘the first country in the western world to implement the statutory regulation of acupuncture under a restriction of title system’. This approach limits use of the Acupuncturist title to specifically-endorsed professionals, but leaves the clinical usage of acupuncture needles in the public domain. Prior to these regulations coming into effect, first in the province of Victoria and subsequently nationwide, *acupuncture* terminology was common among Australian physiotherapists and chiropractors (as well as others) who used fine needles in treating patients. Thereafter, according to Janz and Adams, *dry needling* nomenclature came into widespread usage, both among clinicians and by businesses offering short, acupuncture-related training programs.

In 2013, after statutory title protection was implemented nationwide for acupuncturists, the Physiotherapy Board of Australia advised in a public statement:

‘The restriction on the use of the title ‘acupuncturist’ does not mean that physiotherapists currently practising acupuncture, dry needling or other techniques involving the use of needles need to cease those modalities.’

[73, p. 1]

Other professions, such as occupational therapy, took a similar, if not more explicit stance soon thereafter:

‘The restriction on the use of the title ‘acupuncturist’ does not mean that occupational therapists who practise acupuncture or dry needling can’t continue to use these techniques. However, they may not state that they are performing acupuncture or advertise or hold themselves out to be acupuncturists…’

[89, p. 1]

Drawing attention to an increased number of serious adverse events associated with acupuncture needling, the Chiropractic Board of Australia released a statement in 2014, (and again, almost identically in 2016), that reads:

‘A few National Boards and insurers have noted an unusual increase of pneumothorax [lung puncture] arising from the use of dry needling or acupuncture needles around the thoracic and cervicothoracic areas. While the incidence of such events is still rare, practitioners who are using such needle-based therapies should be:

- aware of this risk and take appropriate steps to both prevent its occurrence in the first instance, and
- able to identify and refer patients with this adverse event for urgent medical care.’ [90, p. 1]

We are unaware of any significant initiatives to contest the policy frameworks surrounding the use of acupuncture needles by chiropractors or physiotherapists in Australia today. However, these two professions are governed by the same regulatory authority as Australia’s Chinese medicine practitioners, which may deter explicit policy challenges by the latter profession. That being said, Australia’s Chinese medicine regulator has ‘been active’ but substantially unsuccessful in attempts use “court action to restrict other practitioners from the therapeutic insertion of needles’ [91, p. 6].

### Part III. Discourses surrounding acupuncture-needling standards for chiropractors and physiotherapists

Across the texts and interviews analysed in this study, we found two primary competing discourses at play with respect to acupuncture needling standards for chiropractors and physiotherapists. We characterize these competing discourses using the phrases *Substantial Distinctiveness and Provider Proficiency* on one hand and, on the other, as *Substantial Equivalence and Patient Safety*. Predominantly representing chiropractors and physiotherapists, one set of stakeholders tended to deploy the first discourse to advocate for relatively brief postgraduate ‘dry needling’ trainings. On the other side of the debate, acupuncturist and physician stakeholders, as well as some physiotherapy and chiropractic organizations, engaged the second discourse to argue that such short trainings represent a significant safety threat to patients. We now detail these competing discourses, providing verbatim quotes to exemplify the type of argumentation identified across the texts and interviews analysed.

#### Discourse I. Substantial distinctiveness and provider proficiency

On one side of the debate, stakeholders argued that the needling practices of chiropractors and physiotherapists differed substantially from the practice of traditional acupuncture, and thus warranted distinct standards of training (which we term a *substantial distinctiveness* discourse). They further asserted that chiropractors and physiotherapists, as experts in treating musculoskeletal disorders, were already proficient in most knowledge and skills needed for the clinical use of filiform needles; as such, short trainings in what they term *dry needling* were sufficient to produce safe, effective practitioners (a *provider proficiency* discourse). This particular discourse is more frequently made explicit among physiotherapists than chiropractors, and in documents originating in the United States rather than in Canada or Australia.

The argumentation involved in this discursive pattern typically proceeds in steps, beginning with an epistemological differentiation of dry needling from traditional acupuncture. For example, in an educational resource paper, the American Physical Therapy Association asserts:

‘The performance of modern dry needling by physical therapists is based on western neuroanatomy and modern scientific study of the musculoskeletal and nervous system. Physical therapists that perform dry needling do not use traditional acupuncture theories or acupuncture terminology. Similarities do exist in terms of dermal penetration with a solid filament needle (a tool) to varying depths within the body for therapeutic indications.’

[92, p. 5]

As this excerpt makes clear, little emphasis is placed in this discourse on the common treatment tool engaged across the needling approaches applied, nor on the needle insertion and manipulation techniques applied. Moreover, as the following excerpt from a book on dry needling demonstrates, no exclusionary jurisdictional claims are made as to which occupational groups may perform the practice:

‘Dry needling is a treatment technique practiced around the globe by numerous healthcare disciplines, including allopathic, osteopathic, naturopathic, podiatric, veterinary, and also chiropractic medicine, acupuncture, physical therapy, dentistry and massage therapy, among others.’

[3, p. 59]

Finally, as demonstrated in a statement by the Irish Society of Chartered Physiotherapists, those arguing for dry needling’s *substantial distinctiveness* from traditional acupuncture generally recognize that dry needling approaches are diversified among its practitioners:

There are varying conceptual models including, but not limited to, superficial dry needling (SDN), deep dry needling (DDN) and intramuscular stimulation (IMS).

[40, p. 9]

In summary, the claim of a biomedical theoretical basis for the clinical use of filiform needles by physiotherapists and chiropractors is centralized, in this discourse, as the essential factor differentiating the practice from traditional acupuncture.

On this basis, the second portion of this discourse, focused on *provider proficiency*, begins with an implicit proposition that since the practices of traditional acupuncture and dry needling are substantially dissimilar, existing training guidelines for the former are irrelevant standards for the latter. The discourse then proceeds more explicitly with a claim that standard professional trainings for all chiropractors and physiotherapists already address a majority of the needed knowledge and skills for safe and effective dry needling practice. This assertion is for instance evident in a position statement by the Alaska State Board of Chiropractic Examiners, which states:

‘Certainly Chiropractic Physicians possess the background and practice skills, with proper training and experience to safely and adequately deliver dry needling services. Chiropractic Physicians are outstanding in anatomy and palpation skills, with a much greater level of training in diagnosis and neurology than a vast majority of professions currently practicing dry needling.’

[93, p. 4]

The Federation of State Boards of Physical Therapy in the United States similarly asserts:

‘More than four-fifths (86%) of what [physical therapists] need to know to be competent in dry needling is acquired during the course of their clinical education.’

[43, p. 13]

Therefore, the proficiency discourse commonly concludes, a relatively short postgraduate training is required to prepare chiropractors and physiotherapists to safely and effectively use filiform needles in their clinical work. In a peer-reviewed paper on the subject, its authors—a physiotherapist and medical doctor—for instance write:

‘The sites for needle insertion are located in skeletal muscles taught in any basic anatomy course. Dry needling is easy to learn, and a basic course usually lasts 2 to 4 days.’

[94, p. 641]

To substantiate the appropriateness of such relatively short post-graduate trainings, the proficiency discourse at times contains brief reference to evidence of safe needling practice among physiotherapists and chiropractors with short trainings. In a submission to the Arizona Board of Physical Therapy, a practitioner-member of that state’s Physical Therapy Dry Needling Task Force asserts:

‘[A] very recently published article …shows that there were no significant adverse events in 7,629 adverse needling treatments offered by physical therapists. The risk of a significant adverse event for dry needling by PTs was calculated to be 0.04%, which is considerably lower than the risk of taking ibuprofen.’

[95, p. 19]

The ‘substantial distinctiveness and provider proficiency’ discourse outlined above appears to significantly drive existing policy formation in the United States, and to a lesser degree in Australia and Canada, with respect to the training standards implemented by chiropractic and physiotherapy regulators. However, this same discourse is also explicitly contested, as we now discuss, by health care providers and policy makers from across the occupational spectrum in a debate that may be characterized as binary, with minor nuances within the opposing subgroups.

#### Discourse II. Substantial equivalence and patient safety

Representing another set of perspectives on this debate, a range of stakeholders from several professions argue that regardless of its epistemic underpinnings, dry needling (and other needling approaches performed by physiotherapists and chiropractors) represents a subset of the traditional acupuncture skillset, therefore warranting similar training. These stakeholders, who represent licensed acupuncturists, physicians, some chiropractic and physiotherapy organizations, and acupuncture educators from across several professions, contend that brief postgraduate trainings were notably insufficient, and represented a significant safety threat to patients. In what we term a *substantial equivalence* discourse, such arguments—when put forth by acupuncturists trained in East Asian medicine—are typified by claims of cultural and medical misappropriation. Such claims argue that physiotherapists and chiropractors are using alternate terminology to advantageously mask their adoption of a treatment approach that has long-standing roots in East Asian medicine. For instance, the American Association of Acupuncture and Oriental Medicine states:

‘Trigger point dry needling and intramuscular manual therapy are aliases used in the marketing of a subset of acupuncture techniques described in the field of acupuncture as “ashi point needling”.’

[79, p. 2]

The American Society of Acupuncturists similarly asserts:

‘“Dry needling” is a pseudonym for acupuncture that has been adopted by physical therapists, chiropractors, and other health providers who lack the legal ability to practice acupuncture within their scope of practice. This strategy allows these groups to skirt safety, testing and certification standards put into place for the practice of acupuncture.’

[77, p. 1]

Other organizations, representing physicians as well as some subgroups of physiotherapists and chiropractors, similarly argue that there is significant overlap between dry needling and acupuncture but do not refer to issues of misappropriation. The American Board of Chiropractic Acupuncture for example states:

‘While both Acupuncture and Dry Needling (DN) are practiced successfully throughout the world, various types of practitioners define dry needling differently. …Every style of needling put forward by “dry needlers” is part of some form of traditional acupuncture practice. …On the other hand, medical, osteopathic and chiropractic physicians may consider dry needling as Western Style Acupuncture or Trigger Point Acupuncture.’

[82, p. 1]

Again echoing the position taken among traditional acupuncture organizations, the Physiotherapy Acupuncture Association of New Zealand suggests that the use of *dry needling* terminology in some jurisdictions amounts to political opportunism:

‘There may be a rationale as to why some countries have embarked on calling the technique ‘dry needling’. This may be due to a restriction of some professions from being able to ‘practise’ acupuncture.’

[26, p. 111]

Such a substantial equivalency discourse, evident across texts representing several health professions, forms the foundation of a safety-related discursive strand. This safety discourse (which differs somewhat between acupuncturists and other regulated professions) characterizes filiform needling standards that fall below the WHO’s training guidelines, as enabling unsafe practice. The American Academy of Medical Acupuncture, an organization of acupuncture-practising physicians, for instance asserts:

‘Regardless of the theory, it is incontrovertible that dry needling is an invasive procedure. Needle length can range up to 4 inches in order to reach the affected muscles. It is critical to understand that dry needling, in the hands of minimally educated practitioners, can cause extreme harm.’

[75, p. 1]

The board of the American Medical Association, the major professional organization of physicians in the United States, similarly asserts in a policy and media statement:

‘Physical therapists and other non-physicians practicing dry needling should—at a minimum—have standards that are similar to the ones for training, certification and continuing education that exist for acupuncture. … Lax regulation and nonexistent standards surround this invasive practice. For patients’ safety, practitioners should meet standards required for licensed acupuncturists and physicians.’

[74, p. 2]

Almost all stakeholders putting forth such a view agree that physiotherapists and chiropractors should be held, at a minimum, to the 200-hour WHO standard for medical doctors’ adjunct practice of acupuncture. Some (though not all) traditional acupuncture organizations and traditional acupuncture educators, however, call for such providers to complete full (that is, 3-year) traditional acupuncture trainings if they are to use filiform needles with patients. This proposition appears predicated on a view at odds with the WHO’s stance that acupuncture may be advantageously learned and practiced as an ‘adjunct’ modality within a biomedical clinical context.

Acupuncture educators from across several professions (including chiropractic and physiotherapy) who were interviewed in our study *universally* agreed that all regulated health professionals who use acupuncture needles should be required at a minimum to meet the WHO’s 200-hour acupuncture training guideline. Further nuancing the safety-related comments evident across the policy statements analysed, these acupuncture educators alluded to their own experiences working within regulatory frameworks in which training requirements for some acupuncture-needling practitioners were left at the individual practitioners’ discretion. One educator who trained both traditional and biomedical acupuncturists describes a common scenario in which physiotherapists enrol in, but do not complete, the course she teaches:

“Physios that take our program don’t have to take the full program to practice. They don’t have to write the exams. They can take one or two courses, and then start practicing. We teach on the Sunday, they can practice on Monday. … I think that you’re doing the public a great disservice by not establishing certain criteria for education and training. I think that’s negligent on the part of the [regulators] to just allow that to happen.”

A biomedical acupuncture educator, familiar with such a trend, similarly argues that brief trainings represent a safety risk to patients:

“One seminar is not safe. Two seminars are not safe. Three seminars. …It doesn’t matter if you’re a doctor, or what profession you come from, you can’t learn acupuncture with one weekend seminar. That’s ridiculous. No, you’re not doing good acupuncture. … Their [regulator] should police them. In most of the world, it’s accepted that 200 hours of teaching in acupuncture is a safe curriculum.”

Another biomedical acupuncture educator calls not only for 200-hour training standards across the professions, but also for a related practical examination:

“I’m an examiner for [name of acupuncture institute] so I examine across the country. We have about thirty percent fail every exam. These people have studied for it, they’ve spent [money] for this exam, they’ve travelled here, and we have a thirty percent failure rate still. So these people who think they’re competent, there’s thirty percent of them that are not competent, because they can’t pass this basic safety exam. … I think everybody in the province, if you’re going to practice acupuncture, should have to do that exam.”

Emphasizing a competing tension between competency and some providers’ wish to quickly complete trainings in the absence of a robust understanding of what constitutes sufficient knowledge, yet another biomedical acupuncture educator asserts:

“It’s the most difficult thing to teach foundational knowledge because all the regulated health professionals, they don’t want that. They’re practitioners and they want fast track, they want to do it on Monday morning.”

This practitioner went on to characterize ‘foundational knowledge’ as aligned with the WHO’s 200-hour standard.

As we now discuss, the two competing discourses presented above are diametrically opposed as to what constitutes sufficient post-graduate training for physiotherapists and chiropractors using acupuncture needles, raising significant policy considerations.

## Discussion and conclusions

Our study findings make evident the wide range of statutory training requirements for the clinical use of filiform needles by physiotherapists and chiropractors across the United States, Canada and Australia. Across these countries, both chiropractors and physiotherapists are permitted to treat musculoskeletal conditions using such needles in a majority of jurisdictions. While a small minority of chiropractic regulators have implemented training standards consistent with the WHO’s 200-hour basic acupuncture guideline, the majority require post-graduate training at or below the 100-hour mark, most frequently without an examination requirement. Across these same jurisdictions, post-graduate training requirements for needling-physiotherapists are yet lower. Whereas a handful of jurisdictions (more so in Canada than elsewhere) require 100 hours of training or more, most physiotherapy regulators either permit individual practitioners to determine what constitutes sufficient training, or stipulate a requirement for less than 100 hours of education.

The terminology used to describe such clinical needling also varies considerably across jurisdictions. Whereas *acupuncture* terminology is dominant among chiropractic and physiotherapy regulators in Canada and within the American chiropractic community, physical therapists across the United States have universally adopted *dry needling* terminology to describe the practice. In Australia, where acupuncture terminology was widespread among physiotherapists and chiropractors prior to acupuncture’s statutory regulation under a restricted title scheme, *dry needling* terminology has more recently become the norm. Similarly, in a few American states and one Canadian province, statutory restrictions on the term *acupuncture* have led chiropractic regulators to widely adopt *dry needling* nomenclature.

The aforementioned training requirements, and terminology choices, have proven significantly controversial across each of the three nations studied. While some occupational groups are undoubtedly adopting opportunistic terminologies to facilitate their clinical usage of acupuncture needles, we take no particular stance here as to which health care professionals be authorized to use acupuncture needles within their clinical scope. Rather, our interest is in the issue of patient safety as it pertains to training requirements in those jurisdictions where physiotherapists and chiropractors *are* permitted to treat patients with acupuncture needles, regardless of the terminology used to describe the practice.

As discussed earlier on, several large quantitative studies have indicated that acupuncture needling—while frequently accompanied by minor adverse effects—may rarely result in severe adverse events such as pneumothorax and even death. There is scientific consensus that acupuncture is a relatively safe therapy when performed by qualified practitioners. However, what constitutes adequate training to ensure the safe clinical use of acupuncture needles by non-physicians who are not licensed acupuncturists remains contested. What our study contributes to this debate is a contextualized qualitative analysis of competing stakeholders’ standards-related claims, in the absence of internationally agreed-upon training or competency guidelines.

In evaluating the discursive claims of various stakeholders, it is important to consider the motivations that may underpin them. As elsewhere demonstrated, including in the context of acupuncture’s professional regulation, stakeholder deployment of safety-related discourses may disproportionately dominate regulatory debates, at times masking a range of important underlying issues warranting attention [see 48]. Such issues may include quality and accessibility of care, professional self-interest, as well as intellectual property and the misappropriation of traditional knowledges. As shown in previous scholarship and emphasized in the present work, this is not to say that safety itself is not of primary significance in professional regulatory negotiation. Rather, it is that risk-related rhetorics may inappropriately draw attention away from other important policy concerns.

We find, furthermore, that discursive claims as to so-called dry needling’s substantial distinctiveness may not hold up under analytic scrutiny, in light of three key points. First, physiotherapists and chiropractors across Canada, the United States and Australia variously use the terms *acupuncture*, *dry needling*, *intramuscular stimulation*, and *meridian therapy*, without a clear indication that there are substantive differences between the practices from place to place where terminology differs. Second, it is evident that in some places, members of the physiotherapy and chiropractic professions are indeed trained in a traditional East Asian approach to acupuncture practice, blurring the boundaries between the needling approaches taken by members of these professions.

Finally, whether or not physiotherapists and chiropractors conceptualize their needling activities biomedically or traditionally holds little relevance to the question of safe training standards for the practice. If the risk associated with the practice has any connection to the tangible needling techniques adopted, consensus that a similar tool is being used in similar tangible ways by diverse practitioners should provide a sufficient basis for common minimum safety standards to be implemented across the professions. Proficiency, or skill in delivering effective treatments, represents an entirely different issue that falls beyond this paper’s scope.

If there is a type of acupuncture with which so-called dry needling may be reasonably compared for standards-related purposes, it is the adjunct practice of acupuncture by medical doctors, many of whom also conceptualize their needling in biomedical (rather than traditional East Asian) terms. It should however be noted that the WHO’s 1999 acupuncture training guidelines took ‘traditional Chinese medical theory…as the basis of [its] Core Syllabus’, even when applied within the broader context of biomedical care [1, p. 3]. At that time, as the WHO noted, important research had begun in which ‘modern Western medical perspectives and research methodologies ha[d] been applied to studies of this traditional therapy’. However, at that time, ‘a new theoretical system ha[d] not yet been established’ for the practice which took biomedical principles as foundational.

Regardless, we concur with Zhou and colleagues [96] that the time is ripe for establishment of a new set of international training guidelines regarding the use of acupuncture needles by a range of health professionals. Such a guideline, we suggest, should ideally take into account the range of conceptual approaches to acupuncture in practice worldwide, whether from within traditional or biomedical epistemologies or both. The guidelines should explicitly differentiate acupuncture needling practices geared to treating a wide range of health disorders (traditional acupuncture, Western medical acupuncture), as well as musculoskeletal conditions (e.g., dry needling) and mental health conditions (e.g., NADA protocols). In addition, this guideline should address the growing trend in several countries—including Canada, the USA and Australia—towards professional regulatory models that permit overlapping (rather than exclusive) scopes of practice [97].

Such a future guideline, which might be undertaken at the WHO level or otherwise, should also take into account the many acupuncture-related conceptual advances in biomedical research since 1999; and specify the degree to which East Asian medical theory may or may not be relevant to various practitioners’ trainings. In addition, such future standards should explicitly take into account the increasing use of filiform needles as adjunct clinical tools by allied and complementary medicine professionals. Finally, it will also be important to address whether or not professionals with pre-existing expertise of some types (e.g., treating musculoskeletal disorders) may require less training in some key aspects of needling therapy. There may also be areas of knowledge in which some professionals may need specific additional training beyond that required of others. Of course it will be incumbent on regulators across various jurisdictions to apply such guidelines with reference to their particular local policy conditions and structures, which may vary considerably.

In its 1999 guidelines, the WHO cautioned against the opportunistic commercialization of acupuncture-related trainings that they characterized as globally widespread. This concern, as our analysis shows, has been similarly raised by a range of stakeholders advocating for established acupuncture standards across the professions. Our study suggests that such opportunism may represent a significant public health issue in some industrialized countries, as suggested in Australian reports indicating a possible increase in pneumothorax incidence that coincides with the relatively unregulated practice of so-called dry needling in that country [90].

Warranting further examination are the intellectual property claims around substantial distinctiveness and equivalency put forth on either side of the debate. Moving beyond considerations of regulatory jurisdiction, traditional acupuncture practitioners’ assertion that dry needling may constitute cultural misappropriation is of particular importance in light of recent United Nations’ recommendations advising regulators to take steps to prevent the misappropriation of traditional medical knowledges. What constitutes cultural poaching versus sharing. hybridity, innovation and evolution in health care context is a complex issue needing further exploration, particularly in light of the WHO’s recent inclusion of *complementary and integrative medicine* under a policy rubric that previously addressed *traditional medicine* more exclusively [98]. However, these important issues fall beyond the scope of the current work’s narrow focus on the concept of safety as it pertains to statutory training standards.

It is clear that acupuncture needles, as clinical tools, can produce serious health outcomes—and policy makers should attend more pointedly to these risks within their distinct regulatory frameworks. Across the jurisdictions studied, many regulators are electing to confer responsibility for determining what constitutes adequate acupuncture-related training on individual practitioners, who may be motivated by educational expediency, or may not be knowledgeable enough about a practice to accurately assess their own skills. By doing so, regulators inappropriately place the burden of risk on the increasing number of patients treated with acupuncture needles across the industrialized world.

Our set of study interviewees did not include educators involved in the type of ‘brief’ postgraduate needling trainings advocated by some stakeholders, which is a limitation of the present study design. Our document analysis clearly identified the primary argumentation at play on both sides of the standards-related debate. That being said, within the Ontario (Canada) context, where study interviews took place, such brief trainings are notably rare (although it is clear that some health care professionals enrol in but only complete portions of longer acupuncture trainings). The ‘instrumental’ generalization of the Ontario case to other English-speaking jurisdictions may be reasonable in that it substantially echoes and triangulates trans-jurisdictional data from across the other jurisdictions studied. However, additional interview data from other jurisdictions, and in particular from educators in the United States and Australia where such brief postgraduate trainings are more commonplace, would be advisable in future to interrogate or affirm the present study’s findings.

Beyond acupuncture, this work points to standards-related tensions that may increasingly arise in light of the recent (but understudied) trend towards overlapping—rather than exclusive—health professional practice scopes in industrialized countries [97, 99]. The movement towards overlapping professional scopes—also evident in drug prescribing and administration authority being granted to multiple professions in a single jurisdiction—has been argued to ‘expand access to care’ by enabling a range of health care providers to ‘play a greater role in meeting increased primary health care demand’ [100, p. 1972]. In the process of future standard setting, it is critical that patient safety remain paramount for regulators. To what degree independent, international training guidelines for particular professional competencies will prove supportive in this regard remains to be seen, and represents an important area for further investigation.

## Supporting information

S1 Table Semi-structured interview guide. (DOCX)
