# Supplementary material for: Evaluating the international standards gap for the use of acupuncture needles by physiotherapists and chiropractors: A policy analysis
Source: PLoS One. 2019 Dec 17;14(12):e0226601. doi: 10.1371/journal.pone.0226601 (PMC6917269; doi:10.1371/journal.pone.0226601)
Supplement: S1 Table — (DOCX) [file pone.0226601.s001.docx]

**S1 Table**

*Note: Interviews in the proposed study were semi-structured in character, in order to accommodate the significant diversity of experiences, perspectives and roles played by various key informants in relation to the regulation of acupuncture in Ontario. What follows is a broad framework outlining key areas of interest explored in interviews (rather than ‘exact’ questions). Some subject areas were be emphasized, minimized or even excluded in particular interviews, in relation to informants’ particular identities and relationships with the study issues. This interview guide underwent appropriate adaptations from informant to informant, while remaining focused on the study themes.*

- - - 1. **OVERALL INVOLVEMENT:** Can you tell me about your involvement in the regulation of acupuncture in Ontario?
         1. *PROBE: Timeframe, particular role, reason for involvement*
         2. *PROBE: Details, story of involvement*
      2. **TCM-BASED & BIOMEDICAL ACUPUNCTURE:** Ontario’s regulations allow for the practice of two broad categories of acupuncture: TCM-based and anatomical, or biomedical, acupuncture. What is your view on how the regulations have handled this issue?
         1. *PROBE: What role do you think culture should or should not play in regulating acupuncture?*
      3. **SCOPE:** Eleven different professional groups in the province are now authorized to perform acupuncture under the new regulations. What is your view on this policy decision?
         1. *PROBE: Personal involvement*
         2. *PROBE: Benefits, challenges associated with this policy*
         3. *PROBE: Economics, legitimation, insurance coverage*
      4. **STANDARDS:** Various professions in Ontario currently require different acupuncture training standards for their members. I am interested in your views on this.
         1. *PROBE: How does your profession handle acupuncture standards?*
         2. *PROBE: Personal involvement in standards development?*
         3. *PROBE: Do you think these standards are adequate? Inadequate?*
         4. *PROBE: Anything else you would like to add about this?*
      5. **GRANDPARENTING:** Each profession regulating acupuncture has handled the issue of ‘grandparenting’ existing practitioners in its own way. In your view, has this process been handled well [by your profession/overall]?
         1. *PROBE: Successes, challenges*
         2. *PROBE: Formal training vs. apprenticeship (TCM only)*
         3. *PROBE: Language issues (TCM only)*
      6. **REGULATORY OBJECTIONS:** There have now been two court cases associated with the province’s acupuncture regulations. Have you had any involvement in these?
         1. *PROBE: If so, describe, discuss…*
         2. *PROBE: If not, what are your views on some TCM practitioners’ objections to the current acupuncture regulations?*
      7. **OPEN-ENDED CONCLUSION:** Is there anything else you would like to add about Ontario’s acupuncture regulations are they stand today?
